# Inducible skeletal muscle-specific p53 deletion alleviates high-fat diet-induced insulin resistance by modulating mitochondria-associated membrane in obese mice^☆^

**DOI:** 10.1016/j.redox.2025.103828

**Published:** 2025-08-20

**Authors:** Soyoung Park, Jin Ki Jung, Jung-Yoon Heo, Themis Thoudam, Su-Yeon Jeong, Seok-Hui Kang, Chang-Hoon Woo, Hyoung Chul Choi, In-kyu Lee, Jinmyoung Dan, Jongsoon Lee, Jae-Ryong Kim, So-Young Park

**Affiliations:** a Senotherapy-based Metabolic Diseases Control Research Center, Republic of Korea; b Department of Physiology, College of Medicine, Yeungnam University, Daegu, 42415, Republic of Korea; c Research Institute of Aging and Metabolism at Kyungpook National University, Daegu, 41404, Republic of Korea; d Division of Nephrology, Department of Internal Medicine, College of Medicine, Yeungnam University, Daegu, 42415, Republic of Korea; e Department of Pharmacology, College of Medicine, Yeungnam University, Daegu, 42415, Republic of Korea; f Department of Internal Medicine, School of Medicine, Kyungpook National University, Kyungpook National University Hospital, Daegu, 41944, Republic of Korea; g Department of Orthopedic Surgery, College of Medicine, CHA University, Gumi, 39295, Republic of Korea; h Soonchunhyang Institute of Medi-Bio Science (SIMS) and Department of Integrated Biomedical Science, Soonchunhyang University, Cheonan-si, 31151, Republic of Korea; i Department of Biochemistry and Molecular Biology, College of Medicine, Yeungnam University, Daegu, 42415, Republic of Korea

**Keywords:** p53, Skeletal muscle, Mitochondria-associated membrane, Insulin resistance, Obesity

## Abstract

p53 has been implicated in metabolic regulation, but its role in obesity-induced skeletal muscle insulin resistance remains incompletely understood. This study aimed to determine the functional contribution of skeletal muscle p53 to insulin resistance and mitochondrial dysfunction, particularly in the context of obesity. We demonstrate that inducible, skeletal muscle-specific deletion of p53 (iMp53 KO) significantly improves insulin sensitivity in high-fat diet (HFD)-induced obese mice, with no effect in chow-fed controls. This metabolic improvement was accompanied by enhanced mitochondrial respiration and membrane potential, as well as reduced mitochondrial calcium overload in palmitate-treated C2C12 myotubes. Electron microscopy and immunoblotting revealed a marked reduction in mitochondria-associated membrane (MAM) area and decreased levels of MAM components (IP3R, VDAC, GRP75) in iMp53 KO muscle. Co-immunoprecipitation assays demonstrated physical interactions between p53 and MAM proteins, supporting a role for p53 in promoting MAM formation under obese conditions. Consistently, skeletal muscle from patients with type 2 diabetes exhibited elevated expression of both p53 and MAM markers, with a positive correlation between them. These findings suggest that p53 plays an important role in modulating ER–mitochondrial contacts and mitochondrial homeostasis in skeletal muscle and suggest its contribution to obesity-induced insulin resistance. This study provides new mechanistic insight into the pathological role of p53 in muscle metabolism.

## Highlights

• p53 deletion in skeletal muscle improves insulin resistance in obese mice.
• p53 loss enhances mitochondrial function and reduces MAM formation in muscle.
• Skeletal muscle p53 directly interacts with key MAM complex components.
• p53 and MAM-related proteins are upregulated in diabetic human skeletal muscle.

## Graphical abstract

**Figure:**
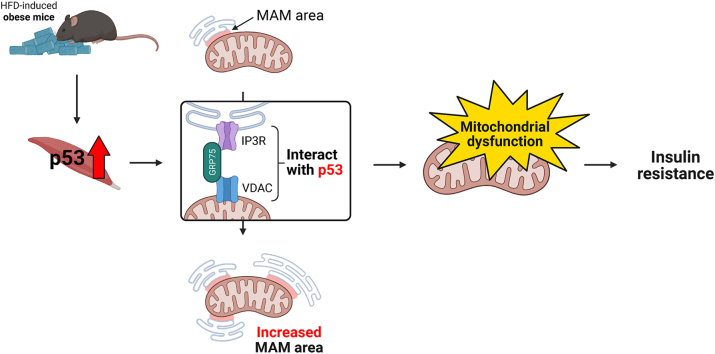

## 1 Introduction

The tumor suppressor p53 plays a multifaceted role, contributing to development, aging, and immune responses [1]. In addition, it regulates several metabolic pathways that are critical for maintaining metabolic homeostasis and suppressing tumorigenesis [2,3]. Notably, its influence on glucose metabolism has been extensively studied, revealing its ability to inhibit malignant progression by suppressing glycolysis and promoting oxidative phosphorylation [2].

Recent evidence also underscores p53's involvement in glucose regulation within insulin-sensitive tissues such as the liver and adipose tissue. Loss of p53 suppresses hepatic glucose production during caloric restriction [4], emphasizing its role in coordinating metabolic adaptation to energy stress. Furthermore, dysregulated p53 expression or activity is linked to metabolic disorders, particularly type 2 diabetes. In patients with type 2 diabetes, hepatic p53 protein levels are elevated and positively correlate with plasma glucose levels and HOMA-IR [4]. Similarly, p53 protein levels are increased in the adipose tissue of diet-induced obese mice, and adipose-specific inhibition of p53 improves insulin resistance in this model [5]. Conversely, overexpression of p53 in adipose tissue induces insulin resistance even under normal chow-fed conditions [5].

Skeletal muscle accounts for approximately 40 % of body weight and is responsible for 70 %–80 % of insulin-stimulated glucose uptake [6,7]. In our previous study, we reported elevated p53 protein levels in the skeletal muscle of aged mice, which were negatively correlated with glucose uptake in this tissue [8]. Furthermore, exercise training was found to reduce p53 expression in the skeletal muscle of type 2 diabetic Goto-Kakizaki rats [9]. These findings suggest an important role for p53 in regulating glucose metabolism and insulin sensitivity in skeletal muscle. However, direct evidence demonstrating whether p53 influences skeletal muscle insulin sensitivity has been lacking.

This study aimed to investigate the effect of p53 deficiency on insulin sensitivity in high-fat diet (HFD)-induced obesity and to elucidate the mechanisms by which p53 regulates insulin signaling in skeletal muscle, using an inducible, skeletal muscle–specific p53 knockout (iMp53 KO) mouse model.

## 2 Materials and methods

### 2.1 Animal studies

All animal experiments were conducted in accordance with protocols approved by the Institutional Animal Care and Use Committee of Yeungnam University College of Medicine (YUMC-AEC2020-036 and YUMC-AEC2024-021). ACTA1-rtTA; tet-O-Cre mice, in which Cre recombinase is inducibly expressed in skeletal muscle upon doxycycline administration, and p53-floxed mice were obtained from the Jackson Laboratory (Bar Harbor, ME, USA). Inducible skeletal muscle-specific p53 knockout (iMp53 KO) mice were generated by crossing p53-floxed mice with ACTA1-rtTA; tet-O-Cre mice. All experiments were conducted using male mice, with p53-floxed littermates serving as controls for comparison with iMp53 KO mice. Both control and iMp53 KO mice were fed either a standard chow diet (2018S, Envigo, Indianapolis, IN, USA) or a 60 % HFD (D12492, Research Diet, New Brunswick, NJ, USA) supplemented with 0.625 g/kg doxycycline, starting at 8 weeks of age for a total duration of 19 weeks. Body weight and food intake were recorded weekly on the same day throughout the feeding period. Mice were housed under a 12-h light/dark cycle with ad libitum access to water. For tissue collection, mice were anesthetized via intraperitoneal injection of Avertin (>1.5 g/kg), and blood was collected from the retro-orbital plexus using heparin-coated capillary tubes. Skeletal muscle tissues were excised, weighed, and either used immediately or snap-frozen and stored at −80 °C.

### 2.2 Intraperitoneal glucose tolerance test (IPGTT) and insulin tolerance test (ITT)

*IPGTT:* Mice were fasted overnight, and body weight was recorded prior to conducting the IPGTT. Baseline blood samples were collected from the tail vein to measure fasting blood glucose and plasma insulin levels. Glucose (1.0 g/kg body weight) was administered intraperitoneally, and blood glucose levels were monitored at 15, 30, 60, 90, and 120 min post-injection using glucose test strips (Roche, Mannheim, Germany). Plasma insulin concentrations were measured at 15, 30, and 60 min using an ELISA kit (Merck Millipore, Kenilworth, NJ, USA) in accordance with the manufacturer's instructions.

*ITT:* For the ITT, mice were fasted for 6 h, and body weight was recorded prior to the procedure. Baseline blood glucose levels were measured from tail vein samples before insulin administration. Human insulin (0.75 U/kg body weight; Eli Lilly and Company, Indianapolis, IN, USA) was administered intraperitoneally, and blood glucose levels were assessed at 15, 30, 60, 90, and 120 min after injection using glucose test strips (Roche).

### 2.3 Tissue collection for insulin signaling pathway analysis

To assess insulin signaling in skeletal muscle, mice were fasted overnight and injected intraperitoneally with insulin at a dose of 1.5 U/kg body weight. After 10 min, muscle tissues were harvested, weighed, and stored at −80 °C until further analysis.

### 2.4 Hyperinsulinemic-euglycemic clamp

Insulin sensitivity was evaluated using the hyperinsulinemic-euglycemic clamp technique, as previously described [10]. During the clamp procedure, human insulin (Eli Lilly and Company) was infused at a rate of 24 pmol kg^−1^ min^−1^ to induce hyperinsulinemia, while plasma glucose levels were maintained at ∼6 mM by a concurrent 20 % glucose infusion. Whole-body glucose turnover was measured by continuous infusion of radiolabeled [3-^3^H] glucose (0.1 μCi/min; PerkinElmer, Waltham, MA, USA), and skeletal muscle glucose uptake was assessed following a bolus injection of 2-deoxy-D-[1–^14^C] glucose (10 μCi, PerkinElmer) at 75 min. Insulin-stimulated hepatic glucose production was calculated by subtracting the glucose infusion rate from the whole-body glucose turnover rate. Plasma glucose levels were determined using a GM9 glucose analyzer (Analox, Stourbridge, UK), and plasma insulin concentrations were measured with an ELISA kit (Merck Millipore), following the manufacturer's instructions. After completion of the hyperinsulinemic-euglycemic clamp, mice were intravenously administered avertin (>1.5 g/kg), and blood samples were collected from the retro-orbital plexus. Tissues were subsequently harvested, weighed, and stored at −80 °C.

### 2.5 Quantitative reverse transcriptase polymerase chain reaction (qRT-PCR)

Gastrocnemius muscles were homogenized in TRIzol (Invitrogen, Waltham, MA, USA), and RNA was extracted from the tissue homogenates using chloroform and isopropanol. The isolated RNA was then reverse transcribed into cDNA using a reverse transcription kit (Applied Biosystems, Waltham, MA, USA). Quantitative real-time PCR (qRT-PCR) was conducted using a Real-Time PCR 7500 System and Power SYBR Green PCR Master Mix (Applied Biosystems), as previously described [10]. The ribosomal protein lateral stalk subunit P0 (Rplp0) served as the reference gene for normalization. The following primers were used: Rplp0 (forward, 5′-CACTGGTCTAGGACCCGAGAA-3′; reverse, 5′-GGTGCCTCTGGAGATTTTCG-3′) and p53 (forward, 5′-ACAGGACCCTGTCACCGAGACC-3′; reverse, 5′-GACCTCCGTCATGTGCTGTGAC-3′).

### 2.6 Western blotting

Skeletal muscle samples were homogenized in lysis buffer containing 50 mM HEPES, 0.22 % β-glycerophosphate, 1 % NP40, 10 % glycerol, 50 mM sodium fluoride, 150 mM NaCl, 1 mM benzamide, 1 mM dithiothreitol, 1 mM sodium orthovanadate, 1 mM phenylmethylsulfonyl fluoride, 1 mM ethylene glycol-bis (β-aminoethyl ether)-N,N,N′,N'-tetraacetic acid, 1 mM EDTA, and a protease inhibitor cocktail (Santa Cruz Biotechnology, Dallas, TX, USA). Tissue homogenates were centrifuged at 16,100×*g* for 10 min at 4 °C, and the resulting supernatant was collected. Protein concentrations were determined using the Bradford assay (Bio-Rad, Hercules, CA, USA). Equal amounts of protein from each sample were separated via sodium dodecyl sulfate–polyacrylamide gel electrophoresis and transferred onto 0.45 μm polyvinylidene fluoride membranes (Merck Millipore). Membranes were blocked with Smart-Block™ 5 min-Fast Blocking Buffer (BIOMAX, Gyeonggi, South Korea) for 5 min, or with 5 % skim milk in Tris-buffered saline containing 0.1 % Tween 20 for 1 h, followed by overnight incubation with primary antibodies at 4 °C. Details of the primary antibodies are provided in Supplementary Table 1. Membranes were then incubated at room temperature for 30 min with secondary antibodies, goat anti-rabbit IgG and goat anti-mouse IgG, both conjugated to horseradish peroxidase (111-035-045 and 111-035-062, Jackson ImmunoResearch, West Grove, PA, USA). Protein detection was carried out using the Fusion Solo S system (Vilber, Eberhardzell, Germany) following treatment with chemiluminescence detection reagents (Merck Millipore). Band intensities were quantified using ImageJ software (National Institutes of Health, Bethesda, MD, USA). Protein levels were normalized to glyceraldehyde 3-phosphate dehydrogenase (GAPDH), and phosphorylated proteins were normalized to their respective total protein levels.

### 2.7 Immunoprecipitation assay

Gastrocnemius muscles were subjected to immunoprecipitation (IP) assays using the Classic Magnetic IP Kit (Thermo Fisher Scientific, #88804, Waltham, MA, USA), following the manufacturer's instructions. Briefly, the gastrocnemius muscle was homogenized in IP Lysis/Wash Buffer supplemented with phosphatase inhibitors using a bead homogenizer (Bioprep-24R; Hangzhou Allsheng Instruments Co., Ltd., Xihu District, Hangzhou, China). The homogenized lysate was centrifuged at 10,000×*g* for 10 min at 4 °C. The resulting supernatant was incubated overnight at 4 °C with rotation in the presence of either control IgG or antibodies specific to the target proteins. Details of the antibodies used are provided in Supplementary Table 1. The antigen–antibody complexes were then incubated with pre-washed magnetic beads at room temperature for 1 h with continuous mixing. After incubation, the beads were collected, the supernatant was discarded, and the beads were washed before eluting the target antigens. Immunoprecipitated proteins were subsequently confirmed via Western blotting. Lysates of Con and KO without immunoprecipitation were substituted as positive controls (input).

### 2.8 Transmission electron microscopy

Tibialis anterior muscles for electron microscopy were prepared as previously described [11]. Mitochondrial and endoplasmic reticulum (ER) morphology was examined using an H-7000 transmission electron microscope (Hitachi, Tokyo, Japan) operated at 75 kV. Mitochondrial counts per unit area and individual mitochondrial areas were randomly measured from three fields per sample, and the mean values were calculated. Mitochondrial area was quantified using ImageJ software (National Institutes of Health, Bethesda, MD, USA). The area of mitochondria-associated membranes (MAMs) was also measured using ImageJ software from at least five randomly selected MAM regions per sample.

### 2.9 Cell culture and shRNA targeting p53

C2C12 mouse myoblasts (ATCC, Manassas, VA, USA) were maintained in Dulbecco's Modified Eagle's Medium (DMEM; WELGENE, Daegu, Korea) supplemented with 10 % fetal bovine serum (Gibco, Amarillo, TX, USA), 100 U/mL penicillin, and 100 μg/mL streptomycin (WELGENE). To induce myogenic differentiation, the medium was replaced with DMEM containing 2 % horse serum (Gibco) and antibiotics. Cells were cultured at 37 °C in a humidified incubator with 5 % CO_2_. For gene knockdown experiments, C2C12 cells were transfected with small hairpin (sh) RNA plasmids targeting either control (shCon; sc-108060) or p53 (shp53; sc-29436-SH) (Santa Cruz Biotechnology) using Lipofectamine 3000 (Thermo Fisher Scientific), following the manufacturer's protocol. A stable cell population was established by selecting transfected cells with 2 μg/mL puromycin 48 h post-transfection**.**

### 2.10 Mitochondrial function

C2C12 myotubes transfected with either shRNA targeting p53 or a control sequence were exposed to 0.25 mM palmitic acid for 12 h. Subsequently, 1.5 × 10^4^ cells per well were plated into an XF-96 cell culture microplate (Agilent Technologies, Cedar Creek, TX, USA). The following day, cells were equilibrated for 1 h at 37 °C in XF DMEM assay medium (Agilent Technologies) supplemented with 25 mM glucose and 4 mM l-glutamine. The oxygen consumption rate (OCR) was then assessed using the XF-96 Analyzer (Agilent Technologies). Sequential injections of oligomycin (1.5 μM), FCCP (1 μM), and a combination of rotenone (0.5 μM) and antimycin A (0.5 μM) were administered to each well. OCR values were normalized to total protein content.

### 2.11 Mitochondrial membrane potential

C2C12 myoblasts transfected with either shRNA targeting p53 or a control sequence were seeded at a density of 1.2 × 10^5^ cells per well in 24-well plates and differentiated in medium containing 2 % horse serum for 5 days. On day 5 of differentiation, cells were treated with 0.25 mM palmitic acid for 12 h. To evaluate mitochondrial membrane potential, JC-1 dye (stock concentration: 7.7 mM in DMSO; Invitrogen) was diluted in serum-free medium and applied to the cells at a final concentration of 10 μM. Cells were incubated at 37 °C for 1 h in the dark and then washed twice with pre-warmed PBS. Green (monomer) and red (aggregate) fluorescence signals were detected using both confocal microscopy (Nanoscope Systems, Daejeon, Korea) equipped with a × 20 objective lens and a fluorescence microplate reader (BioTek, Winooski, VT, USA). For confocal microscopy, excitation/emission wavelengths were set to approximately 488/530 nm for JC-1 monomers and 561/595 nm for JC-1 aggregates. For microplate reader measurements, excitation/emission settings were 485/528 nm for JC-1 monomers and 540/620 nm for JC-1 aggregates. Mitochondrial membrane potential was evaluated based on the red-to-green fluorescence ratio obtained using the fluorescence microplate reader.

### 2.12 Calcium measurement using flow cytometry

Mitochondrial Ca^2+^ levels in C2C12 cells transfected with either shRNA targeting p53 or a control sequence were assessed by Rhod-2 AM fluorescence (R1244; Invitrogen) using both flow cytometry and confocal microscopy, in accordance with the manufacturer's instructions. For flow cytometry analysis, C2C12 myoblasts were seeded at a density of 1.2 × 10^5^ cells per well in 12-well plates and differentiated in DMEM supplemented with 2 % horse serum for 4 days. On day 4, the resulting myotubes were treated with 0.25 mM palmitic acid for 1 or 3 h, washed with PBS, and incubated with 5 μM Rhod-2 AM for 30 min at 37 °C in the dark. Following three PBS washes, stained cells were analyzed by flow cytometry (CytoFLEX, Beckman Coulter, Brea, CA, USA).

For confocal microscopy, C2C12 myoblasts were seeded at a density of 8 × 10^4^ cells per well in 4-well chamber slides and differentiated under the same conditions. Cells were treated with 0.25 mM palmitic acid for 3 h, washed with PBS, and co-stained with 5 μM Rhod-2 AM and 200 nM MitoTracker (M7514; Invitrogen) for 30 min at 37 °C in the dark. After three PBS washes, cells were counterstained with DAPI (1:500) for 10 min and visualized using a laser scanning confocal microscope equipped with a × 20 objective (Nanoscope Systems).

### 2.13 Human muscle sample

Human skeletal muscle samples were collected from the anterior deltoid and middle deltoid with approval from the Institutional Review Boards (IRBs) of Yeungnam University (IRB protocol YU202007011) and Gumi CHA Hospital (IRB protocol GM19-08). All tissue specimens were obtained from Gumi CHA Hospital after obtaining written informed consent. Participant identities were anonymized in accordance with IRB guidelines. Samples were categorized into control or type 2 diabetes groups based on (1) medical history of diabetes and (2) HbA1c levels. Age and BMI did not differ significantly between the two groups (Supplementary Table 2).

### 2.14 Statistical analysis

Statistical analyses were carried out using Prism 8 software (GraphPad Software, San Diego, CA, USA). Differences between groups were evaluated using unpaired two-tailed Student's *t*-tests and one-way analysis of variance (ANOVA), followed by Tukey's honestly significant difference test. Data are reported as mean ± standard error (SE), and p-values less than 0.05 were considered statistically significant.

## 3 Results

### 3.1 p53 is elevated in skeletal muscle of obese mice and patients with type 2 diabetes

**Fig. 1:**
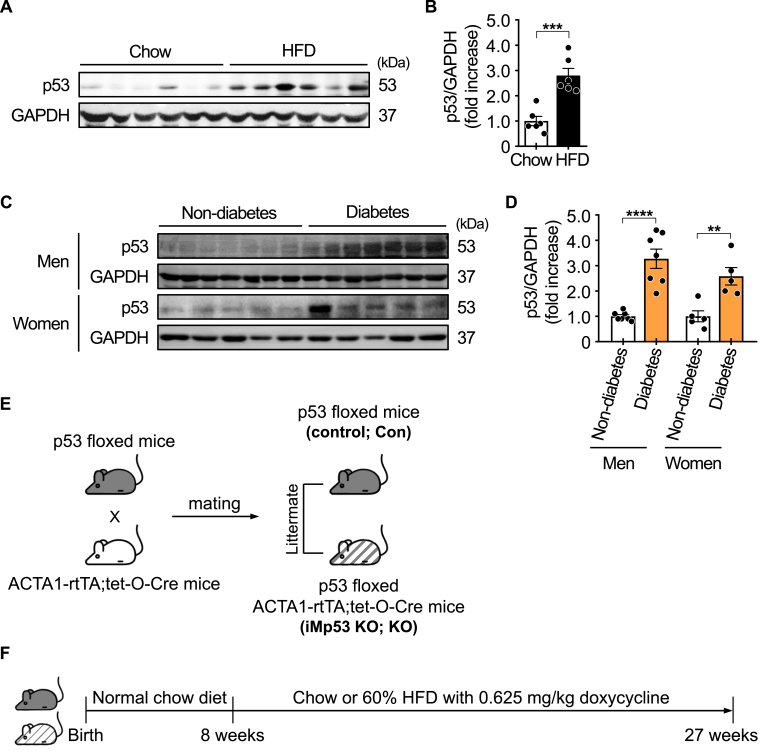
**Skeletal muscle p53 expression under metabolic stress and generation of an inducible skeletal muscle-specific p53 knockout model.** (A–B) p53 protein expression in the gastrocnemius muscle of male mice fed a high-fat diet (HFD) for 12 weeks (*n* = 6 per group). (C–D) p53 protein expression in the deltoid muscle of patients with type 2 diabetes and non-diabetic individuals (*n* = 5-7 per group). (E) Schematic diagram illustrating the generation of the inducible skeletal muscle-specific p53 knockout (iMp53 KO) mouse model. (F) Experimental timeline depicting doxycycline administration and dietary intervention. GAPDH was used as a loading control. All samples were biologically independent. Data are presented as mean ± SE. Statistical analysis was performed using a two-tailed Student's *t*-test for comparisons between two groups. ∗∗*p* < 0.01, ∗∗∗*p* < 0.001, and ∗∗∗∗*p* < 0.0001. Abbreviations: Con, control; GAPDH, glyceraldehyde-3-phosphate dehydrogenase; KO, inducible skeletal muscle-specific p53 knockout.

To assess whether p53 expression is elevated in the skeletal muscle of metabolically compromised individuals, we measured p53 protein levels in skeletal muscle samples from HFD-fed obese mice and patients with type 2 diabetes. p53 protein levels were significantly increased in the gastrocnemius muscle of obese mice fed an HFD for 12 weeks (Fig. 1A and B). In addition, p53 protein expression was markedly elevated in the skeletal muscle of diabetic patients—both men and women—compared with BMI- and age-matched non-diabetic individuals (Fig. 1C and D). Previous studies have shown that p53 mRNA levels in adipose tissue positively correlate with BMI in humans [12], and that hepatic p53 protein levels are significantly elevated in both mouse models and patients with type 2 diabetes [4]. In this study, we present novel evidence indicating that p53 protein is significantly upregulated in the skeletal muscle of obese mice and patients with type 2 diabetes, suggesting a potential role for p53 in the pathophysiology of metabolic diseases in skeletal muscle.

### 3.2 iMp53 deletion improves whole-body insulin resistance in obese mice

To elucidate the metabolic role of p53 in skeletal muscle, we generated iMp53 KO mice by crossing p53 floxed mice with ACTA1-rtTA; tet-O-Cre mice. At 8 weeks of age, the mice were placed on a doxycycline-containing diet, which was maintained throughout the experimental period (Fig. 1E and F).

**Fig. 2:**
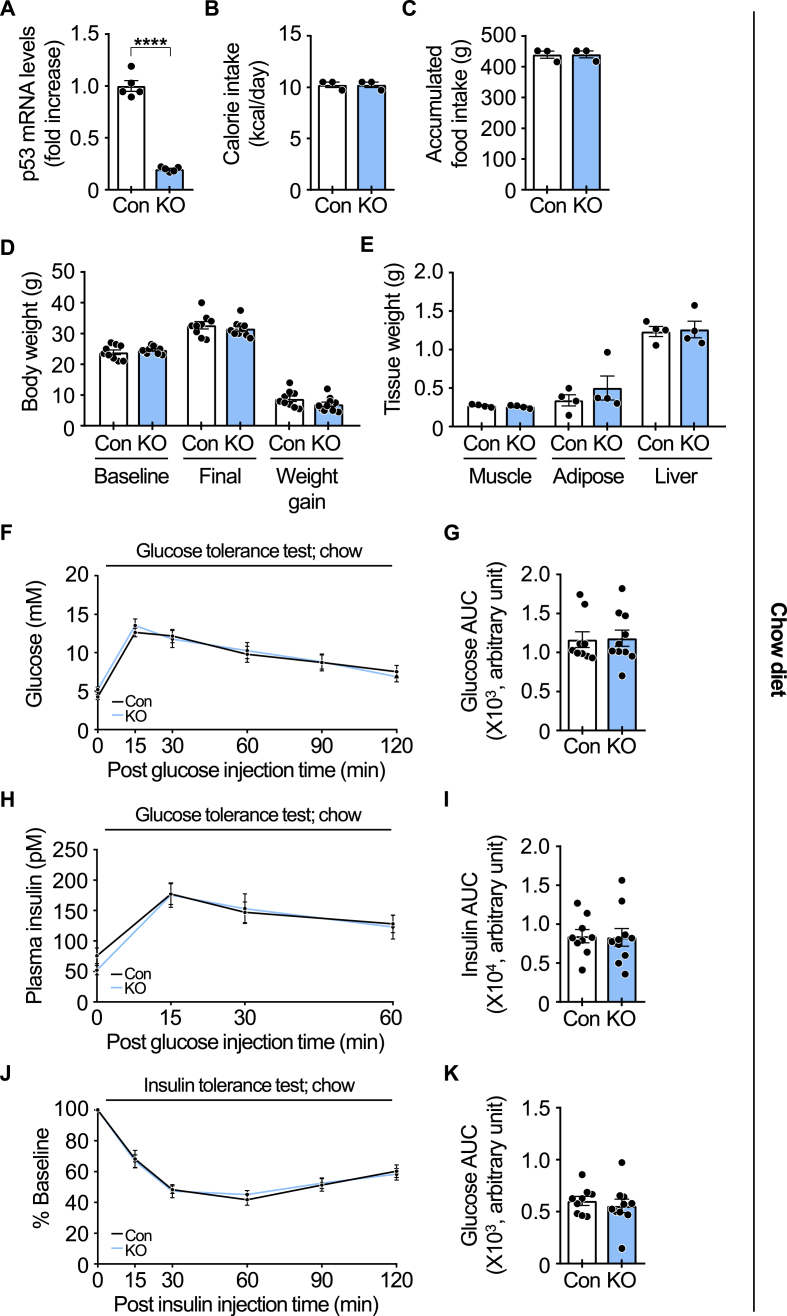
**p53 deficiency does not alter insulin sensitivity under metabolically normal conditions.** Eight-week-old control and iMp53 KO male mice were fed a doxycycline-containing chow diet for 19 weeks. (A) p53 gene expression in the gastrocnemius muscle (*n* = 5 per group). (B) Average daily calorie intake (*n* = 3 per group). (C) Accumulated food intake during the experimental period (*n* = 3 per group). (D) Initial baseline body weight, final body weight, and weight gain during the experimental period (*n* = 9–10 per group). (E) Weights of skeletal muscle, epididymal fat tissue, and liver (*n* = 4 per group). (F–G) Blood glucose levels and area under the curve (AUC) during the glucose tolerance test (*n* = 9–10 per group). (H–I) Plasma insulin levels and AUC during the glucose tolerance test (*n* = 9–10 per group). (J–K) Percent reduction in blood glucose from baseline and AUC during the insulin tolerance test (*n* = 9–10 per group). All samples were biologically independent. Data are presented as mean ± SE. Statistical significance was determined using a two-tailed Student's t-test for comparisons between two groups. ∗∗∗∗*p* < 0.0001. Abbreviations: BW, body weight; Con, control; KO, inducible skeletal muscle-specific p53 knockout.

After 19 weeks on the doxycycline-containing chow diet, p53 mRNA levels were significantly reduced in the gastrocnemius muscle of iMp53 KO mice compared to control mice (Fig. 2A). Deletion of p53 did not affect food intake (Fig. 2B and C), body weight (Fig. 2D), gastrocnemius muscle mass, epididymal fat mass, and liver weight (Fig. 2E). No significant differences were observed in fasting blood glucose or plasma insulin levels between the two groups. Following intraperitoneal glucose administration, blood glucose levels increased similarly in both control and iMp53 KO mice, with no significant differences in either glucose or insulin levels (Fig. 2F–I). Likewise, intraperitoneal insulin injection reduced blood glucose levels, but the response was comparable between groups (Fig. 2J and K). These findings indicate that iMp53 deletion does not influence glucose metabolism or insulin sensitivity in mice fed a standard chow diet.

**Fig. 3:**
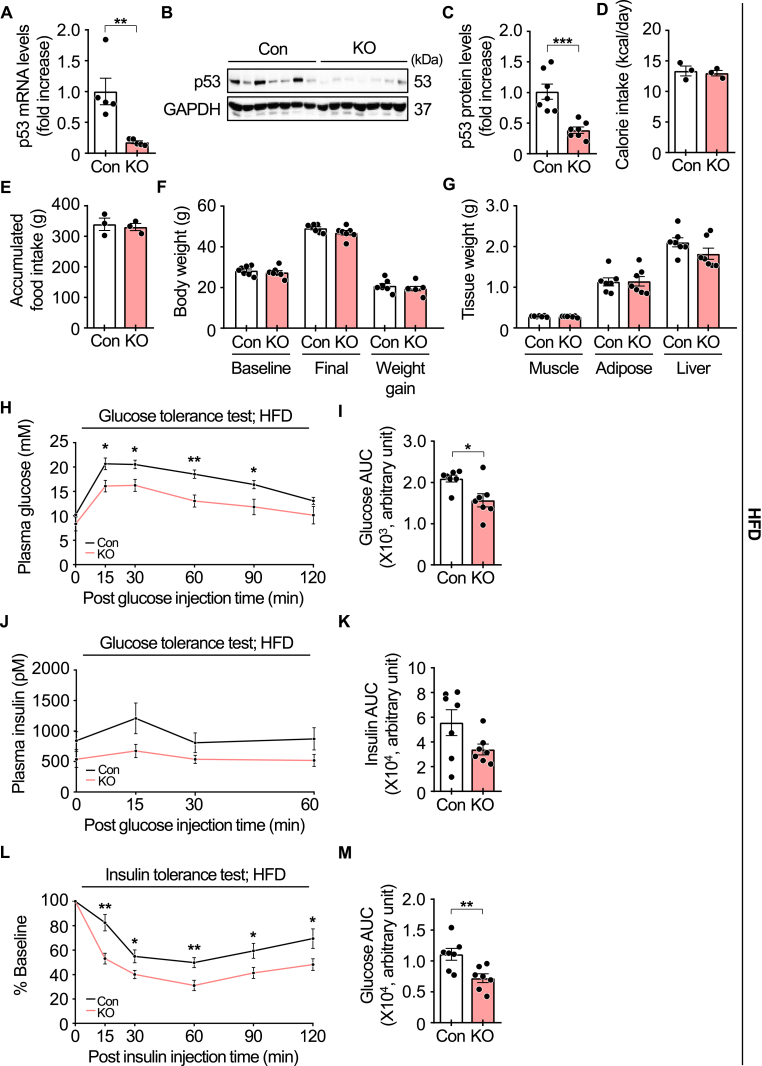
**p53 deficiency improves insulin resistance in HFD-fed obese mice.** Eight-week-old control and iMp53 KO male mice were fed a doxycycline-containing high-fat diet (HFD) for 19 weeks. Data shown in panels H–K were collected after 10 weeks of HFD feeding, while data in panels L and M were obtained after 19 weeks of HFD feeding. (A) p53 gene expression in the gastrocnemius muscle (*n* = 5 per group). (B–C) p53 protein expression in the gastrocnemius muscle (*n* = 7 per group). (D) Average daily calorie intake (*n* = 3 per group). (E) Accumulated food intake during the experimental period (*n* = 3 per group). (F) Initial baseline body weight, final body weight, and weight gain during the experimental period (*n* = 7 per group). (G) Weights of skeletal muscle, epididymal fat tissue, and liver (*n* = 7 per group). (H–I) Blood glucose levels and area under the curve (AUC) during the glucose tolerance test (*n* = 7 per group). (J–K) Plasma insulin levels and AUC during the glucose tolerance test (*n* = 7 per group). (L–M) Percent reduction in blood glucose from baseline and AUC during the insulin tolerance test (*n* = 7 per group). All samples were biologically independent. Data are presented as mean ± SE. Statistical significance was assessed using a two-tailed Student's *t*-test for comparisons between two groups. ∗*p* < 0.05, ∗∗*p* < 0.01, and ∗∗∗*p* < 0.001. Abbreviations: BW, body weight; Con, control; GAPDH, glyceraldehyde-3-phosphate dehydrogenase; KO, inducible skeletal muscle-specific p53 knockout.

Next, we examined the impact of inducible p53 deletion in the skeletal muscle of obese mice. A doxycycline-containing HFD effectively suppressed p53 expression in the gastrocnemius muscle after 19 weeks of feeding (Fig. 3A–C). As observed in chow-fed iMp53 KO mice, food intake, body weight, skeletal muscle mass, epididymal fat mass, and liver weight were not significantly different between control and iMp53 KO mice after HFD feeding (Fig. 3D–G). Fasting blood glucose and plasma insulin levels after 10 weeks of HFD feeding tended to be lower in iMp53 KO mice than in controls. Following intraperitoneal glucose injection, blood glucose levels increased in both groups, but were significantly lower in iMp53 KO mice. Consistently, the area under the curve (AUC) for glucose was also reduced in KO mice (Fig. 3H and I). Plasma insulin levels and insulin AUC similarly tended to be lower in iMp53 KO mice (Fig. 3J and K). An insulin tolerance test (ITT) conducted after 11 weeks of HFD feeding showed no significant difference in percent basal glucose levels between the groups (data not shown). However, a second ITT performed at 19 weeks revealed significantly lower percent basal glucose levels and AUC in iMp53 KO mice compared to controls (Fig. 3L and M). These findings suggest that skeletal muscle-specific inducible deletion of p53 improves glucose intolerance and insulin resistance in diet-induced obese mice.

### 3.3 iMp53 deletion improves skeletal muscle insulin resistance in obese mice

**Fig. 4:**
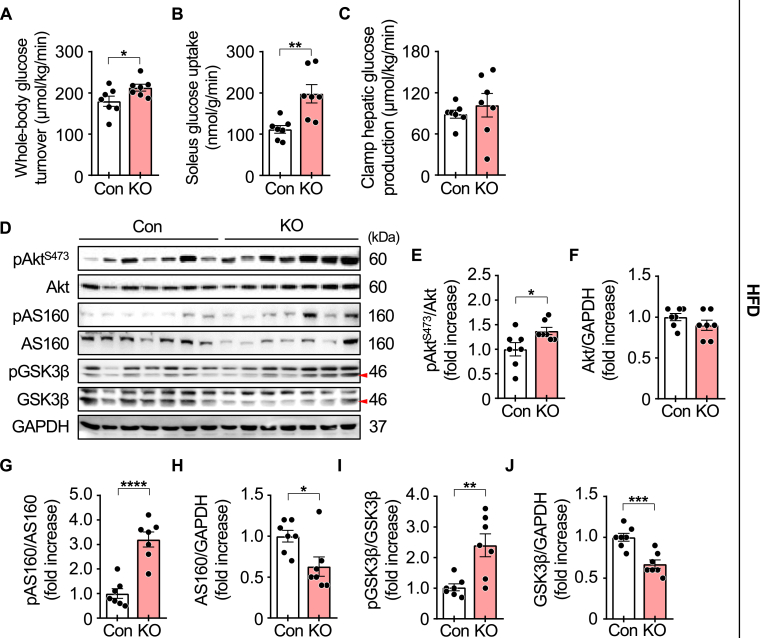
**p53 deficiency enhances insulin-stimulated glucose disposal and signaling in skeletal muscle of HFD-fed obese mice.** Eight-week-old control and iMp53 KO male mice were fed a doxycycline-containing high-fat diet (HFD) for 19 weeks. Following HFD feeding, a hyperinsulinemic-euglycemic clamp test was performed to assess whole-body and tissue-specific insulin sensitivity (A–C; *n* = 7 per group), including whole-body glucose turnover (A), glucose uptake in the soleus muscle (B), and hepatic glucose production (C). To evaluate insulin signaling, mice were fasted overnight and administered insulin (1.5 U/kg) intraperitoneally; gastrocnemius muscles were harvested 10 min post-injection, and insulin signaling proteins were analyzed by Western blot (D–J; *n* = 7 per group). GAPDH was used as a loading control, and phosphorylated proteins were normalized to their respective total protein levels. Representative blots of insulin signaling proteins (D), phosphorylated Akt (pAkt; Ser473) levels (E), total Akt (F), phosphorylated AS160 (pAS160) levels (G), total AS160 (H), phosphorylated GSK3β (pGSK3β) levels (I), and total GSK3β (J). All samples were biologically independent. Data are presented as mean ± SE, and statistical significance was determined using a two-tailed Student's *t*-test for comparisons between two groups. ∗*p* < 0.05, ∗∗*p* < 0.01, ∗∗∗*p* < 0.001, and ∗∗∗∗*p* < 0.0001. Abbreviations: AS160, Akt substrate of 160 kDa; Con, control; GAPDH, glyceraldehyde-3-phosphate dehydrogenase; GSK3β, glycogen synthase kinase 3β; KO, inducible skeletal muscle-specific p53 knockout.

To determine whether skeletal muscle contributes to improved insulin sensitivity in obese mice, we performed a hyperinsulinemic-euglycemic clamp test in HFD-fed mice after 19 weeks of feeding. Plasma glucose levels were maintained at approximately 6.6 mM, while plasma insulin concentrations were 1727.3 ± 57.3 pM in control mice and 1642.2 ± 41.7 pM in iMp53 KO mice during the clamp procedure. Whole-body glucose turnover was significantly higher in iMp53 KO than in control mice (Fig. 4A), and skeletal muscle glucose uptake was also elevated in iMp53 KO mice (Fig. 4B). In contrast, hepatic glucose production did not differ between the two groups (Fig. 4C). These results indicate that inducible p53 deletion enhances insulin sensitivity in skeletal muscle but not in the liver of obese mice.

Insulin binding to its receptor initiates a cascade of downstream signaling events, including activation of the phosphoinositide 3-kinase/Akt/Akt substrate of 160 kDa (AS160) pathway, which promotes the translocation of cytosolic glucose transporter 4 (GLUT4) to the plasma membrane to facilitate glucose uptake [13]. Activated Akt also phosphorylates glycogen synthase kinase 3β (GSK3β), thereby promoting glycogen synthesis [14]. Impaired activation of these insulin signaling pathways is a major contributor to insulin resistance. To validate improved insulin sensitivity in iMp53 KO mice, we assessed the activation status of key signaling proteins. Levels of phosphorylated Akt (Ser473), AS160, and GSK3β were significantly increased in the gastrocnemius muscle of iMp53 KO mice (Fig. 4D–J), supporting the conclusion that skeletal muscle-specific p53 deletion enhances insulin signaling and improves insulin sensitivity in obese mice.

### 3.4 iMp53 deletion improves skeletal muscle mitochondrial function in obese mice

**Fig. 5:**
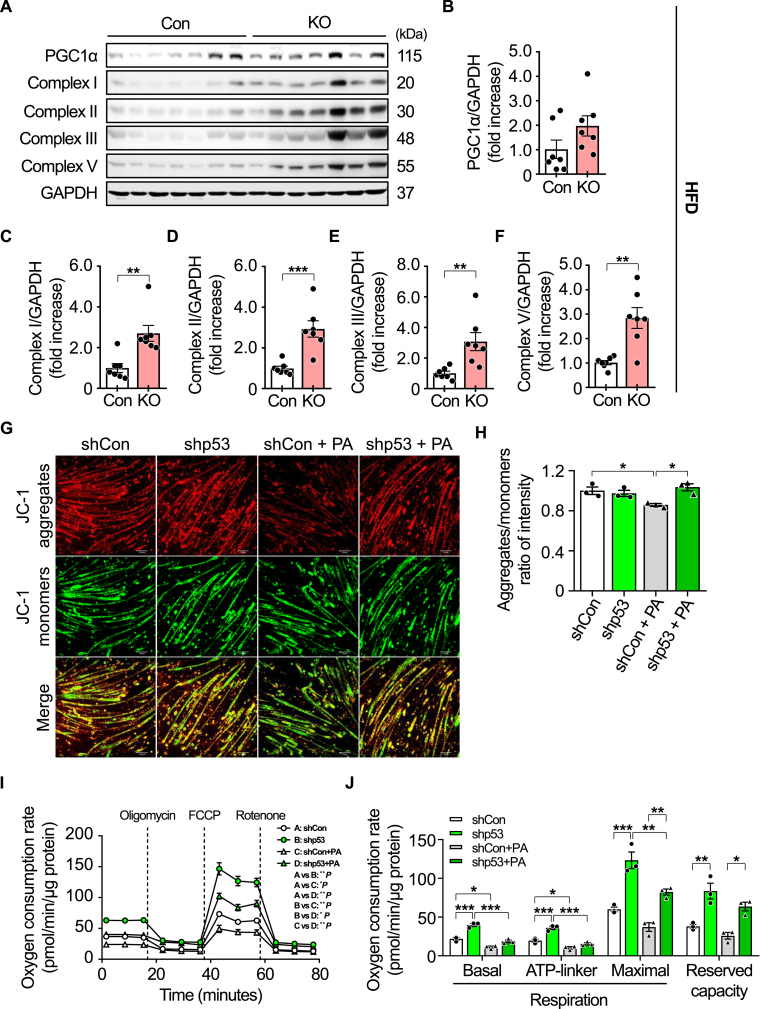
**p53 deficiency improves mitochondrial membrane potential and respiratory capacity.** Eight-week-old control and iMp53 KO male mice were fed a doxycycline-containing high-fat diet (HFD) for 19 weeks. Protein levels of mitochondrial electron transport chain complexes and PGC1α in the gastrocnemius muscle were analyzed by Western blot (A–F; *n* = 7 per group), with GAPDH used as a loading control. Representative Western blots (A) quantification of PGC1α (B) and quantification of mitochondrial electron transport chain complex proteins (C–F). *In vitro*, C2C12 myoblasts were transfected with shCon or shp53, induced to differentiate, and then treated with 0.25 mM palmitic acid for 12 h (G–J). Differentiated myotubes were incubated with 10 μM JC-1 dye for 1 h; fluorescence images were captured using a fluorescence microscope, and signal intensities were quantified using a fluorescence microplate reader (G–H). Mitochondrial membrane potential was assessed by JC-1 fluorescence; representative images are shown (G) and quantification analysis of JC-1 signal (H; *n* = 3 per group). Mitochondrial oxygen consumption rate in C2C12 myotubes was measured using the Seahorse system (I–J; *n* = 3 per group). All samples were biologically independent. Data are presented as mean ± SE. Statistical significance was determined using a two-tailed Student's *t*-test for comparisons between two groups and one-way ANOVA followed by Tukey's post hoc test for comparisons among four groups. ∗*p* < 0.05, ∗∗*p* < 0.01, and ∗∗∗*p* < 0.001. Abbreviations: Con, control; GAPDH, glyceraldehyde-3-phosphate dehydrogenase; KO, inducible skeletal muscle-specific p53 knockout; PA, palmitic acid; PGC1α, peroxisome proliferator-activated receptor gamma coactivator 1 alpha; shCon, control shRNA-transfected C2C12 cells; shp53, p53 shRNA-transfected C2C12 cells.

Mitochondrial dysfunction contributes to the development of insulin resistance in the liver and skeletal muscle of obese individuals [[15], [16], [17]]. To investigate the mechanisms by which iMp53 KO improves insulin resistance in obese mice, we assessed the expression of mitochondrial electron transport chain (ETC) proteins in the gastrocnemius muscle. Inducible deletion of p53 increased ETC protein levels, accompanied by elevated expression of peroxisome proliferator-activated receptor gamma coactivator 1-alpha (PGC-1α) in obese mice (Fig. 5A–F), whereas no differences were observed between control and iMp53 KO mice fed a chow diet (Fig. S1A–F). Additionally, p53 deletion in skeletal muscle did not significantly affect mitochondrial area or number in mice fed a chow diet (Fig. S1G–I). We also confirmed enhanced mitochondrial function in p53-deficient C2C12 myotubes following palmitic acid treatment. Palmitic acid significantly impaired mitochondrial membrane potential, which was restored by p53 knockdown (Fig. 5G and H). Furthermore, p53 deletion increased both maximal respiration and mitochondrial reserve capacity following palmitic acid exposure (Fig. 5I and J). These findings suggest that p53 deletion enhances mitochondrial function in the skeletal muscle of obese mice.

### 3.5 iMp53 deletion reduces MAM formation in the skeletal muscle of obese mice

**Fig. 6:**
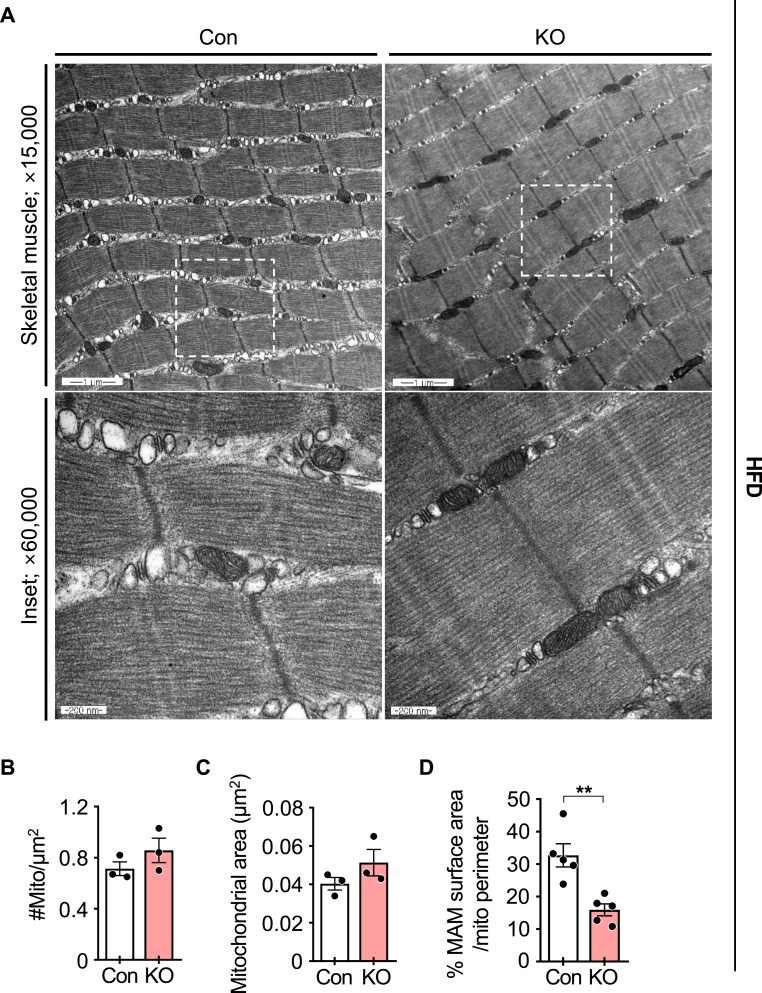
**p53 deficiency reduces the area of mitochondria-associated membranes in skeletal muscle of HFD-fed obese mice.** Eight-week-old control and iMp53 KO male mice were fed a doxycycline-containing high-fat diet (HFD) for 19 weeks. Transmission electron microscopy was used to visualize mitochondria in the tibialis anterior muscle at magnifications of ×15,000 and × 60,000 (inset) (A). Mitochondrial density (number per μm^2^; B) and mitochondrial area (C) were quantified (B–C; *n* = 3 per group). The ratio of mitochondria-associated membrane (MAM) length relative to mitochondrial perimeter was also quantified (D; *n* = 5 per group). All samples were biologically independent. Data are presented as mean ± SE. Statistical significance was determined using a two-tailed Student's *t*-test for comparisons between two groups. ∗∗*p* < 0.01. Abbreviations: Con, control; KO, inducible skeletal muscle-specific p53 knockout.

Recent studies have shown that p53 modulates mitochondrial function by translocating from the cytoplasm to mitochondria, where it localizes to mitochondria-associated membranes (MAMs) [18,19]. MAMs, which are physical contact sites between the endoplasmic reticulum (ER) and mitochondria, play a critical role in calcium transfer from the ER to mitochondria [20,21]. Disruption of MAM integrity has been associated with mitochondrial calcium overload and dysfunction [[22], [23], [24], [25]]. Thus, maintaining MAM structural integrity is essential for proper insulin signaling in both liver and skeletal muscle [[22], [23], [24], [25], [26]]. In the present study, we found that while p53 deletion did not significantly alter mitochondrial number or morphology in chow-fed mice (Fig. S1G–I), it did lead to a reduction in MAM area in the tibialis anterior muscle of obese mice (Fig. 6A–D). This suggests that reduced MAM formation may contribute to improved insulin sensitivity in HFD-fed obese mice.

To investigate the role of p53 in regulating MAMs in skeletal muscle, we analyzed the expression of key MAM-related proteins and sarcoplasmic/endoplasmic reticulum Ca^2+^-ATPase 1 (SERCA1) under conditions of metabolic stress and p53 deficiency. MAMs are composed of three macromolecular complexes. The inositol 1,4,5-trisphosphate receptor type 1 (IP3R1), a calcium-release channel located on the ER, interacts with the voltage-dependent anion channel (VDAC) on the mitochondrial outer membrane through the molecular chaperone glucose-regulated protein 75 (GRP75) [21,27]. SERCA pumps calcium from the cytosol into the sarcoplasmic reticulum [28].

**Fig. 7:**
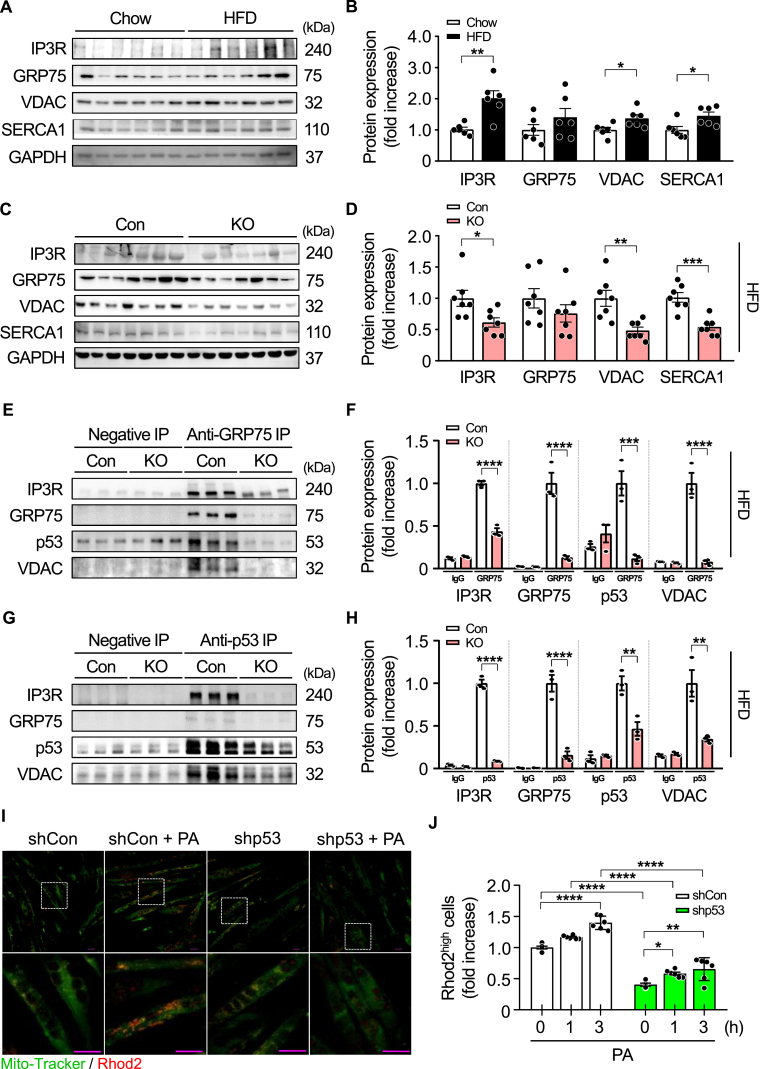
**Skeletal muscle p53 interacts with mitochondria-associated membrane (MAM) components and regulates mitochondrial calcium loading.** Eight-week-old male mice were fed either a chow diet or a high-fat diet (HFD) for 12 weeks, after which expression levels of MAM-related proteins and SERCA1 in the tibialis anterior muscle were analyzed by Western blot (A–B; *n* = 6 per group). Eight-week-old control and iMp53 KO male mice were then fed a doxycycline-containing HFD for 19 weeks, and expression levels of MAM components and SERCA1 were again assessed in the gastrocnemius muscle via Western blot (C–D; *n* = 7 per group). GAPDH was used as a loading control. GRP75-targeted immunoprecipitation was performed using gastrocnemius muscle lysates from control and p53-deficient mice to assess protein–protein interactions (E–F), and p53-targeted immunoprecipitation was conducted to evaluate its interaction with MAM components (G–H; *n* = 3 per group). To assess mitochondrial calcium dynamics, C2C12 cells transfected with either shCon or shp53 were treated with 0.25 mM palmitic acid for 1 h or 3 h on day 5 of differentiation. Mitochondrial calcium levels were visualized via confocal microscopy (I) and quantified by flow cytometry (J; *n* = 6 per group). All samples were biologically independent. Data are presented as mean ± SE. Statistical significance was determined using a two-tailed Student's *t-*test for comparisons between two groups and one-way ANOVA followed by Tukey's post hoc test for comparisons among four or more groups. ∗*p* < 0.05, ∗∗*p* < 0.01, ∗∗∗*p* < 0.001, and ∗∗∗∗*p* < 0.0001. Abbreviations: Con, control; KO, inducible skeletal muscle-specific p53 knockout; GAPDH, glyceraldehyde-3-phosphate dehydrogenase; GRP75, glucose-regulated protein 75; IP3R, inositol 1,4,5-trisphosphate receptor; PA, palmitic acid; SERCA1, sarcoplasmic reticulum Ca^2+^ATPase 1; shCon, control shRNA-transfected C2C12 cell; shp53, p53 shRNA-transfected C2C12 cell; VDAC, voltage-dependent anion channel.

HFD feeding significantly increased the protein levels of IP3R, VDAC, and SERCA1 in the tibialis anterior muscle compared to chow-fed controls (Fig. 7A and B), indicating enhanced MAM formation in response to metabolic stress. In contrast, inducible deletion of p53 markedly reduced the expression of IP3R, VDAC, and SERCA1 in the gastrocnemius muscle (Fig. 7C and D) and soleus muscle (Fig. S2A–E) under HFD conditions.

These findings suggest that p53 contributes to the upregulation of MAM components in diet-induced obese mice.

To confirm the reduction of MAMs in iMp53 KO mice, we performed immunoprecipitation (IP) analyses targeting key MAM-associated proteins. IP with *anti*-GRP75 antibodies in gastrocnemius muscle lysates revealed significantly lower GRP75 levels in iMp53 KO mice compared to controls-, along with reduced expression of IP3R, VDAC, and p53, indicating that p53 deficiency mitigates aberrant MAM formation (Fig. 7E and F). Additional IP analyses using *anti*-IP3R and *anti*-VDAC antibodies further confirmed reduced expression of MAM-associated proteins in iMp53 KO mice (Fig. S3A–D). These results suggest a physical association between p53 and MAM components in skeletal muscle of obese mice.

We performed IP analyses using *anti*-p53 antibodies to confirm the localization of p53 at MAMs. Skeletal muscle from iMp53 KO mice exhibited a marked reduction in p53 expression, accompanied by decreased levels of IP3R, GRP75, and VDAC (Fig. 7G and H), supporting the hypothesis that p53 physically interacts with MAM components and plays a critical role in promoting MAM formation in obese mice.

Next, to determine whether p53 deletion alleviates mitochondrial calcium overload under metabolic stress, we performed Rhod-2 staining combined with MitoTracker in C2C12 myotubes treated with palmitic acid. In control cells, palmitic acid markedly increased mitochondrial Rhod-2 fluorescence, indicating calcium overload, whereas this response was significantly attenuated by p53 knockdown (Fig. 7I). Flow cytometric analysis further confirmed a time-dependent reduction in the Rhod-2^high^ population in p53-deficient myotubes (Fig. 7J). These results suggest that p53 deficiency in skeletal muscle suppresses mitochondrial calcium overload by limiting MAM formation during metabolic stress.

### 3.6 The expression of MAM components is higher in the skeletal muscle of diabetic patients

**Fig. 8:**
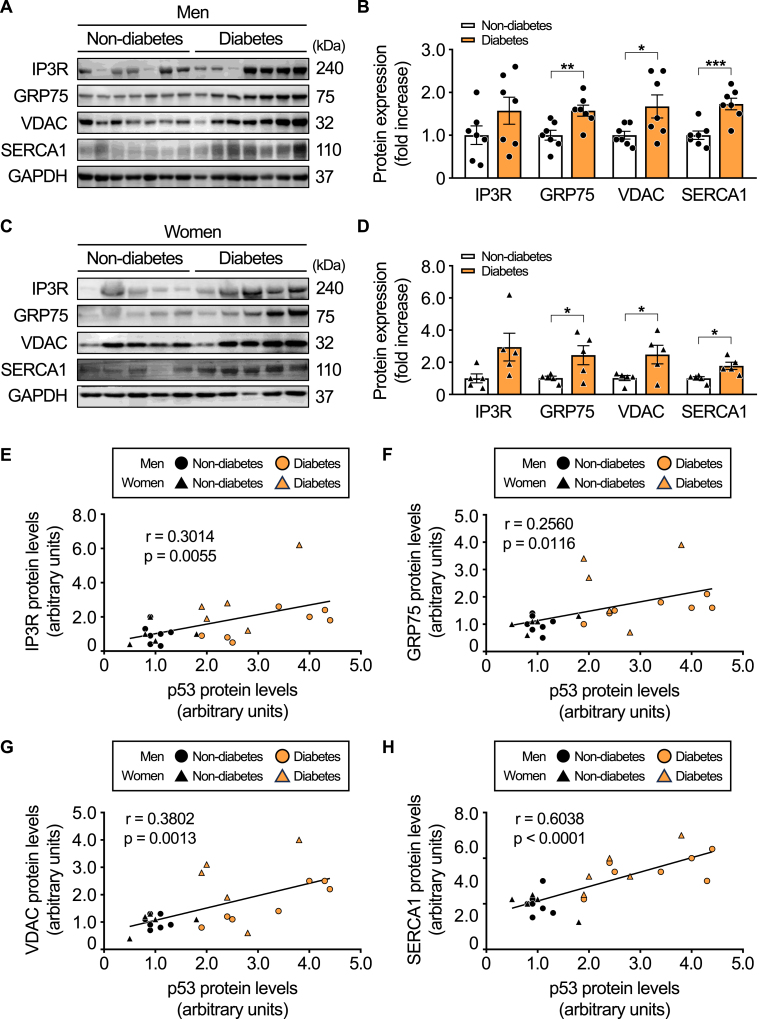
**p53 protein expression is positively correlated with mitochondria-associated membrane (MAM)-related proteins in human skeletal muscle.** Protein expression levels of IP3R, GRP75, VDAC, and SERCA1 were analyzed in the deltoid muscle samples from non-diabetic and diabetic individuals (A–D; *n* = 5-7 per group), with GAPDH used as a loading control. Correlations between p53 protein levels and the expression of MAM-related proteins were assessed across all human skeletal muscle samples (E–H; *n* = 24). All samples were biologically independent. Data are presented as mean ± SE. Statistical significance was determined using a two-tailed Student's *t*-test for comparisons between two groups. ∗*p* < 0.05, ∗∗*p* < 0.01, and ∗∗∗*p* < 0.001. Abbreviations: GAPDH, glyceraldehyde-3-phosphate dehydrogenase; GRP75, glucose-regulated protein 75; IP3R, inositol 1,4,5-trisphosphate receptor; SERCA1, sarcoplasmic reticulum Ca^2+^-ATPase 1; VDAC, voltage-dependent anion channel.

To evaluate whether these findings in obese animal models are reflected in humans, we analyzed the expression levels of key MAM-associated proteins and SERCA1 in skeletal muscle biopsies from patients with type 2 diabetes and non-diabetic individuals. Age and BMI were matched between the two groups. Protein levels of GRP75, VDAC, and SERCA1 were significantly elevated in the skeletal muscle of patients with type 2 diabetes compared to non-diabetic controls, in both men and women, indicating enhanced MAM formation under diabetic conditions (Fig. 8A–D).

To further assess whether the relationship between p53 and MAM-related proteins observed in obese mice also exists in human metabolic disease, we analyzed correlations between p53 and MAM component expression in skeletal muscle samples from individuals with and without diabetes. p53 expression positively correlated with IP3R, GRP75, VDAC, and SERCA1 protein levels in human skeletal muscle (Fig. 8E–H), suggesting that p53 may contribute to enhanced MAM formation in the context of diabetes.

## 4 Discussion

In the present study, we investigated the metabolic role of p53 in skeletal muscle under obese conditions using a skeletal muscle-specific, inducible p53 knockout mouse model. Our findings demonstrate that deletion of p53 in skeletal muscle significantly improves insulin sensitivity and enhances mitochondrial function in obese mice, accompanied by a reduction in MAM formation. Co-immunoprecipitation assays further support a potential physical interaction between p53 and MAM components, suggesting a direct role for p53 in promoting MAM assembly under obese conditions. Additionally, skeletal muscle samples from patients with type 2 diabetes exhibited elevated levels of p53 and MAM components, with a positive correlation observed between p53 and MAM-related proteins in human skeletal muscle. These findings suggest a pathophysiological role for p53 in regulating mitochondrial function and contributing to insulin resistance in human skeletal muscle.

HFD induces both oxidative stress and inflammation, leading to diverse metabolic disturbances, including disrupted glucose metabolism, impaired mitochondrial function, hepatic steatosis, and metabolic changes in the brain and adipose tissue [[29], [30], [31]]. Previous studies have shown that HFD increases p53 expression in adipose tissue and liver, which promotes insulin resistance by enhancing the transcriptional activity of phosphoenolpyruvate carboxykinase 1 and promoting inflammation [4,5]. In the current study, we extend these findings to skeletal muscle, demonstrating that HFD increases p53 expression in skeletal muscle and muscle-specific deletion of p53 ameliorates diet-induced insulin resistance, at least in part, by suppressing aberrant MAM formation, an event known to impair mitochondrial function and disrupt insulin signaling.

Although p53 has been implicated in regulating mitochondrial biogenesis and function in skeletal muscle, prior studies report conflicting outcomes, particularly depending on the specificity of gene deletion. Global p53 knockout results in abnormal mitochondrial morphology along with reduced mitochondrial content and function [32,33]. In contrast, constitutive skeletal muscle-specific deletion of p53 does not appear to affect mitochondrial content or oxidative capacity under basal conditions [[34], [35], [36]].

Consistent with previous findings, we also demonstrate that muscle-specific p53 knockout does not affect mitochondrial number, mitochondrial area, or the expression of ETC complexes in the skeletal muscle of chow diet-fed mice. The discrepancy between global and tissue-specific knockout models remains unresolved, warranting further investigation. Nevertheless, these results suggest that p53 is dispensable for maintaining basal mitochondrial homeostasis in skeletal muscle.

Interestingly, while a previous study reported exacerbated mitochondrial dysfunction in skeletal muscle-specific p53 knockout mice under stress conditions such as denervation [35], our findings reveal the opposite effect under HFD-induced metabolic stress. Specifically, we observed increased expression of ETC complexes and PGC-1α in p53-deficient skeletal muscle following HFD feeding, indicating enhanced mitochondrial function. This beneficial effect of p53 deletion is further supported by our observation that it improves insulin resistance in obese mice. A key distinction lies in the experimental models: previous studies used a constitutive knockout approach, where p53 was deleted in skeletal muscle from the embryonic stage. In contrast, our study employed an inducible system, enabling p53 deletion specifically in adult muscle. The reason these differing models yield opposing outcomes remains unclear and warrants further investigation to uncover the underlying mechanisms. Nevertheless, our inducible model offers a more physiologically relevant context for examining p53 inhibition, as it isolates the metabolic role of p53 in adult skeletal muscle without confounding developmental adaptations. It is also plausible that p53 exerts context-dependent effects, acting as a positive regulator of mitochondrial quality control under certain stress conditions (e.g., disuse or injury), while inhibiting mitochondrial expansion during metabolic overload.

The improved mitochondrial function observed in obese iMp53 KO mice in this study may be attributed, at least in part, to a reduction in MAM area. Prior studies have demonstrated that an HFD promotes MAM formation in both liver and skeletal muscle, contributing to mitochondrial calcium overload, dysfunction, and insulin resistance [22,24]. In agreement with our findings, reducing MAM area or limiting mitochondrial calcium transport in obese mice has been shown to improve mitochondrial oxidative capacity and enhance insulin sensitivity in metabolic tissues [22,24].

Excessive 10.13039/100027893MAM formation and the resulting disruption of calcium signaling have been implicated in the pathogenesis of insulin resistance [24,37]. Supporting this, we show that p53 deletion in C2C12 myotubes significantly reduces mitochondrial calcium accumulation. Based on these results, we propose that HFD-induced upregulation of p53 may drive MAM expansion, leading to mitochondrial calcium imbalance and promoting insulin resistance.

Although the precise mechanisms by which p53 promotes MAM formation remain to be elucidated, our data suggest that p53 physically interacts with at least one key MAM component. While p53 may be dispensable for the initial assembly of MAMs, it could contribute to the stabilization of MAM structure or regulate its functional properties under metabolic stress conditions such as obesity.

A limitation of this study is that we did not assess oxidative stress markers in iMp53 KO mice. Given that mitochondrial dysfunction is closely associated with oxidative stress [38], it is plausible that the improved mitochondrial function observed in iMp53 KO mice may attenuate oxidative stress. Incorporating oxidative stress markers would further strengthen our conclusions regarding the role of p53 in regulating mitochondrial function in skeletal muscle. In conclusion, our findings demonstrate a regulatory role of p53 in the mitochondrial function in skeletal muscle under metabolic stress. While not essential for maintaining basal mitochondrial homeostasis, p53 contributes to MAM expansion and mitochondrial calcium overload in obesity and diabetes. The consistent elevation of p53 and MAM-related proteins in the skeletal muscle of diabetic patients, along with mechanistic evidence from mouse and cell models, supports the notion that p53 links metabolic stress to mitochondrial dysfunction and insulin resistance by modulating MAM structure and function. By connecting p53-dependent MAM expansion to mitochondrial Ca^2+^ overload and impaired insulin action in skeletal muscle, these findings identify the p53–MAM axis as a novel therapeutic target. Limiting p53-driven MAM expansion may improve metabolic health in obesity and type 2 diabetes.

## CRediT authorship contribution statement

**Soyoung Park:** Writing – original draft, Visualization, Validation, Resources, Methodology, Investigation, Funding acquisition, Formal analysis, Conceptualization. **Jin Ki Jung:** Writing – original draft, Visualization, Validation, Methodology, Investigation, Formal analysis. **Jung-Yoon Heo:** Writing – original draft, Validation, Investigation, Formal analysis. **Themis Thoudam:** Investigation. **Su-Yeon Jeong:** Investigation. **Seok-Hui Kang:** Validation, Resources. **Chang-Hoon Woo:** Writing – review & editing, Validation, Resources. **Hyoung Chul Choi:** Resources, Methodology. **In-kyu Lee:** Validation, Resources. **Jinmyoung Dan:** Writing – review & editing, Validation, Resources. **Jongsoon Lee:** Writing – review & editing, Validation, Funding acquisition. **Jae-Ryong Kim:** Writing – review & editing, Validation, Resources. **So-Young Park:** Writing – review & editing, Writing – original draft, Project administration, Methodology, Funding acquisition, Conceptualization.

## Funding

This work was supported by the 10.13039/501100003725National Research Foundation of Korea (NRF) grant funded by the Korea 10.13039/501100014188Ministry of Science and ICT (MSIT) [NRF-2020R1A6A3A13077496, 2021R1A5A808014513, 2022R1A5A2018865].

## Declaration of competing interest

The authors declare that they have no known competing financial interests or personal relationships that could have appeared to influence the work reported in this paper.

## Data Availability

Data will be made available on request.

## Abbreviations

**ANOVA**: analysis of variance
**AS160**: Akt substrate of 160 kDa
**AUC**: area under the curve
**BW**: body weight
**Con**: control
**ER**: endoplasmic reticulum
**ETC**: electron transport chain
**GAPDH**: glyceraldehyde-3-phosphate dehydrogenase
**GLUT4**: glucose transporter 4
**GRP75**: glucose-regulated protein 75
**GSK3β**: glycogen synthase kinase 3β
**HFD**: high-fat diet
**iMp53 KO**: inducible skeletal muscle-specific p53 knockout
**IP**: immunoprecipitation
**IP3R**: inositol 1,4,5-trisphosphate receptor
**IPGTT**: intraperitoneal glucose tolerance test
**IRBs**: institutional review boards
**ITT**: insulin tolerance test
**KO**: inducible skeletal muscle-specific p53 knockout
**OCR**: oxygen consumption rate
**PA**: palmitic acid
**PGC1α**: peroxisome proliferator-activated receptor gamma coactivator 1 alpha
**qRT-PCR**: quantitative reverse transcriptase polymerase chain reaction
**Rplp0**: ribosomal protein lateral stalk subunit P0
**SE**: standard error
**SERCA1**: sarcoplasmic reticulum Ca^2+^ATPase 1
**sh**: small hairpin
**shCon**: control shRNA-transfected C2C12 cells
**shp53**: p53 shRNA-transfected C2C12 cells
**VDAC**: voltage-dependent anion channel
