# Supplementary material for: Inducible skeletal muscle-specific p53 deletion alleviates high-fat diet-induced insulin resistance by modulating mitochondria-associated membrane in obese mice
Source: Redox Biol. 2025 Aug 20;86:103828. doi: 10.1016/j.redox.2025.103828 (PMC12540039; doi:10.1016/j.redox.2025.103828)
Supplement: Multimedia component 1 [file mmc1.docx]

**
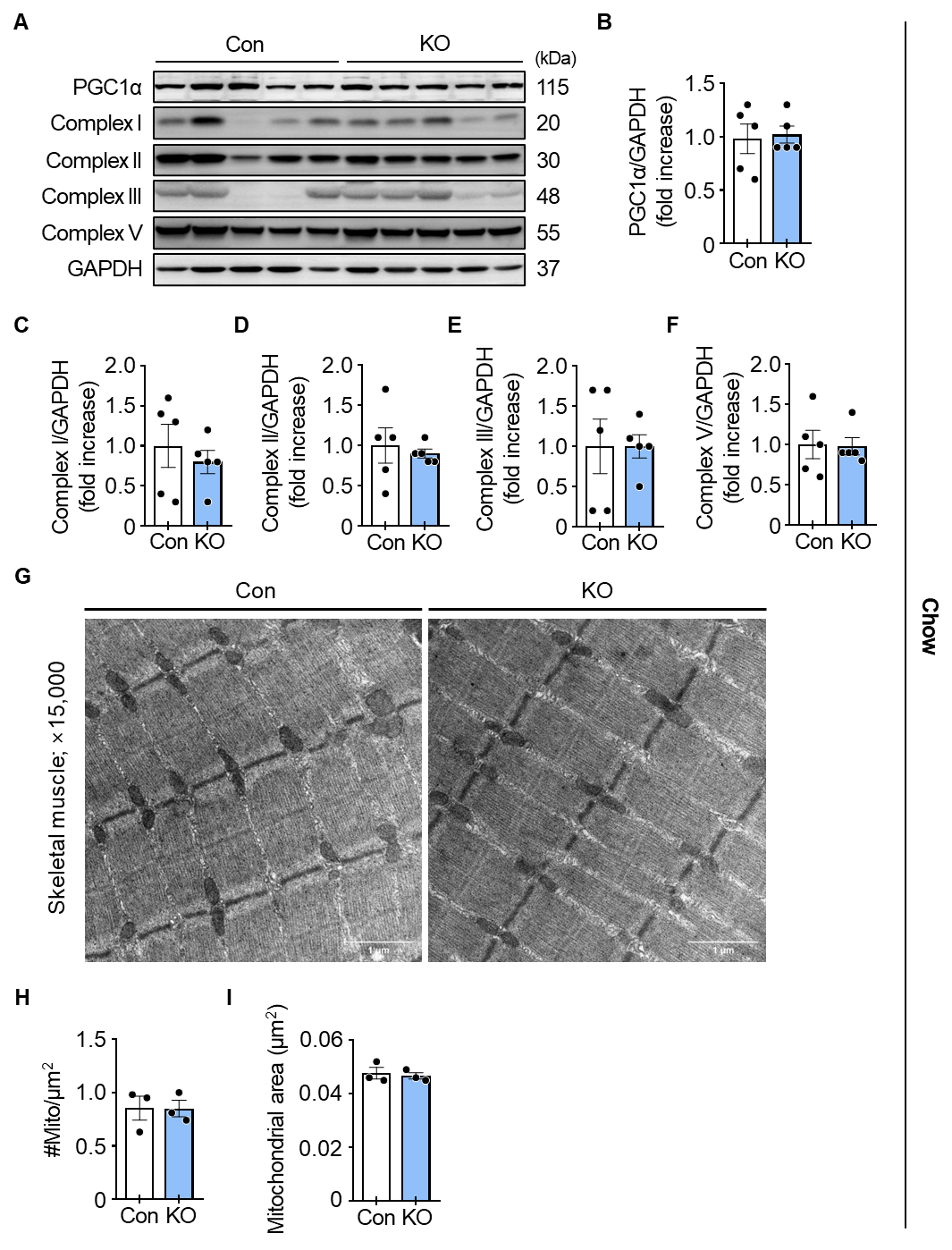
Supplementary Figures and Legends**

**Supplementary Figure 1. p53 deficiency does not alter mitochondrial contents or expression of mitochondrial proteins in chow-fed mice**. Eight-week-old control and iMp53KO male mice were fed a doxycycline-containing chow diet for 19 weeks. (A–F) Protein levels of PGC1α and mitochondrial electron transport chain complexes in the gastrocnemius muscle were assessed by Western blot (A–F; n = 5 per group), with GAPDH used as a loading control. (G–I) Transmission electron microscopy (×15,000 magnification) images of mitochondria in tibialis anterior muscle, with quantification of mitochondrial number per μm^2^ and mean mitochondrial area (n = 3 per group). All samples were biologically independent. Data are presented as mean ± SE. Statistical significance was determined using a two-tailed Student’s t-test for comparisons between two groups. Abbreviations: Con, control; GAPDH, glyceraldehyde-3-phosphate dehydrogenase; KO, inducible skeletal muscle-specific p53 knockout; PGC1α, peroxisome proliferator-activated receptor gamma coactivator 1 alpha.


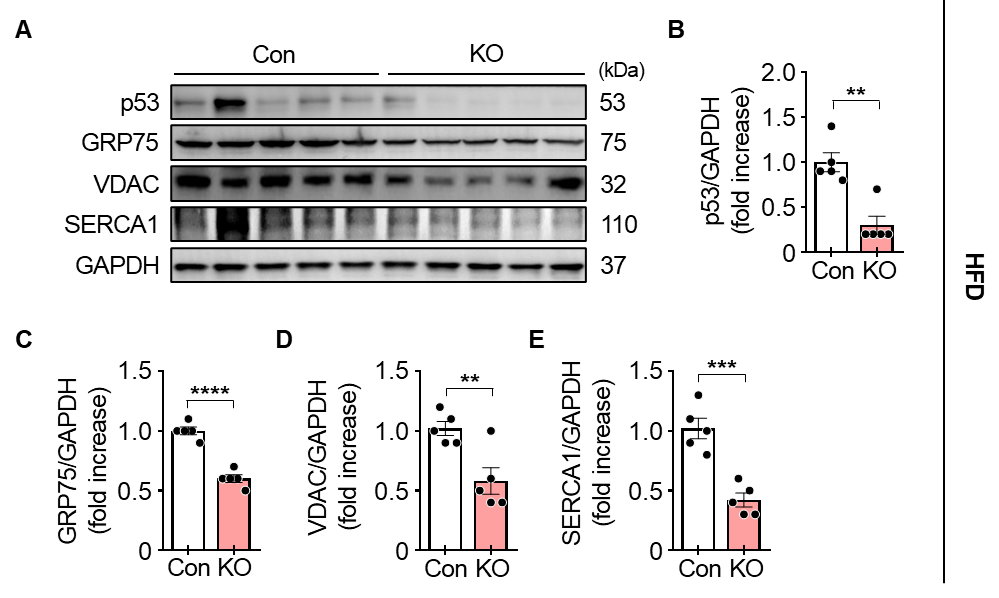


**Supplementary Figure 2. p53 deficiency reduces the mitochondria-associated membrane (MAM) components in skeletal muscle of HFD-fed mice**. Eight-week-old control and iMp53KO male mice were fed a doxycycline-containing high-fat diet (HFD) for 19 weeks. (A–E) Protein levels of MAM components and SERCA1 in the soleus muscle were assessed by Western blot (n = 5 per group), with GAPDH used as a loading control. All samples were biologically independent. Data are presented as mean ± SE. Statistical significance was determined using a two-tailed Student’s t-test for comparisons between two groups. ***p* < 0.01, ****p* < 0.001, and *****p* < 0.0001. Abbreviations: Con, control; GAPDH, glyceraldehyde-3-phosphate dehydrogenase; GRP75, glucose-regulated protein 75; KO, inducible skeletal muscle-specific p53 knockout; SERCA1, sarcoplasmic reticulum Ca2+ATPase 1; VDAC, voltage-dependent anion channel.


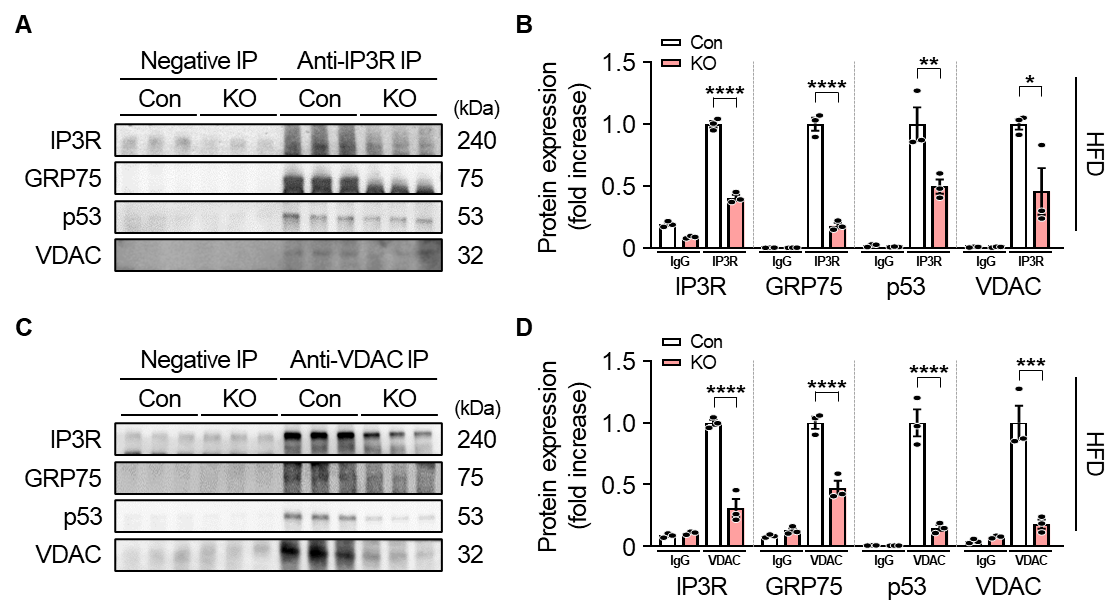


**Supplementary Figure 3. p53 deficiency downregulates the IP3R**–**GRP75**–**VDAC complex in skeletal muscle of HFD-fed mice.** Eight-week-old control and iMp53KO male mice were fed a doxycycline-containing high-fat diet (HFD) for 19 weeks. Protein–protein interactions were assessed by immunoprecipitation in skeletal muscle using either IP3R-targeted (A–B) or VDAC-targeted (C–D) approaches (*n* = 3 per group). All samples were biologically independent. Data are presented as mean ± SE. Statistical significance was determined using one-way ANOVA followed by Tukey’s post hoc test for comparisons among the four groups. **p* < 0.05, ***p* < 0.01, ****p* < 0.001, and *****p* < 0.0001. Abbreviations: Con, control; GRP75, glucose-regulated protein 75; IP3R, inositol 1,4,5-trisphosphate receptor; KO, inducible skeletal muscle-specific p53 knockout; VDAC, voltage-dependent anion channel.

**Supplementary Table 1.** List of primary antibodies used in the experiments.

| **Antibodies** | **Technology** | **Catalog number** | **Application** |
| --- | --- | --- | --- |
| p53 | Santa Cruz Biotechnology | #sc-126 | Western blotting |
| p53 | Cell Signaling Technology | #2524 | Immunoprecipitation |
| pAkt (Ser473) | Cell Signaling Technology | #9271 | Western blotting |
| Akt | Cell Signaling Technology | #9272 | Western blotting |
| pAS160 | Cell Signaling Technology | #8881 | Western blotting |
| AS160 | Cell Signaling Technology | #2670 | Western blotting |
| pGSK3β | Cell Signaling Technology | #8566 | Western blotting |
| GSK3β | Cell Signaling Technology | #5676 | Western blotting |
| PGC1α | Sigma-Aldrich | #516557 | Western blotting |
| Complex I | Abcam | #ab110413 | Western blotting |
| Complex II | Abcam | #ab110413 | Western blotting |
| Complex III | Abcam | #ab110413 | Western blotting |
| Complex V | Abcam | #ab110413 | Western blotting |
| IP3R | Santa Cruz Biotechnology | #sc-377518 | Western blotting |
| IP3R | Cell Signaling Technology | #8568 | Immunoprecipitation |
| GRP75 | Cell Signaling Technology | #3593 | Western blotting  /Immunoprecipitation |
| VDAC1 | Santa Cruz Biotechnology | #sc-390996 | Western blotting  /Immunoprecipitation |
| SERCA1 | Abcam | #ab2819 | Western blotting |
| Normal rabbit IgG | Cell Signaling Technology | #2729 | Immunoprecipitation |
| Normal mouse IgG | Santa Cruz Biotechnology | #sc-2025 | Immunoprecipitation |
| GAPDH (mice) | Santa Cruz Biotechnology | #sc-32233 | Western blotting |
| GAPDH (human) | Santa Cruz Biotechnology | #sc-47724 | Western blotting |

**Supplementary Table 2.** Demographic and clinical information of human participants included in the study.

|  | **Non-diabetes** | **Diabetes** | ***P* value** |
| --- | --- | --- | --- |
| Number of cases (F)* | 12 (5) | 12 (5) |  |
| Age (yr) | 63.8 ± 1.09 | 65.4 ± 1.41 | 0.3587 |
| Duration of diabetes (mo) | 0 | 39.3 ± 10.97** | 0.0017 |
| Fasting blood glucose (mM) | 5.6 ± 0.15 | 7.9 ± 0.65 | 0.0018 |
| Hb A1c (%) | 5.4 ± 0.07 | 6.7 ± 0.23 | 0.000017 |
| Hb A1c (mmol/mol) | 34.9 ± 0.90 | 49.2 ± 2.46 | 0.000017 |
| Weight (kg) | 64.1 ± 2.51 | 65.6 ± 2.98 | 0.6899 |
| Height (cm) | 164.5 ± 2.67 | 161.9 ± 2.60 | 0.4960 |
| BMI (kg/m^-2^) | 23.6 ± 0.56 | 24.9 ± 0.58 | 0.1321 |
| Systolic blood pressure (mmHg) | 116.7 ± 2.84 | 121.7 ± 2.71 | 0.2160 |
| Diastolic blood pressure (mmHg) | 72.5 ± 1.79 | 75.8 ± 2.59 | 0.3133 |

*The number of female participants (F) in brackets.

**Two people were diagnosed with diabetes when they visited Gumi Cha Hospital.
Therefore, the duration of diabetes is not accurate.
